# Organizational climate, job satisfaction, and burnout in nursing workers

**DOI:** 10.47626/1679-4435-2022-867

**Published:** 2023-08-08

**Authors:** Mirian Cristina dos Santos Almeida, Vinicius Gomes Barros, Silmar Maria da Silva, Fábio José da Silva, Ricardo Toshio Yamassake, Anna Cláudia Mauricio Telles, Renan Sallazar Ferreira Pereira, Patricia Campos Pavan Baptista

**Affiliations:** 1 Departamento de Enfermagem, Universidade Federal de Tocantins, Palmas, TO, Brazil; 2 Programa de Pós-Graduação em Gerenciamento em Enfermagem, Escola de Enfermagem, Universidade de São Paulo (USP), São Paulo, SP, Brazil; 3 Departamento de Enfermagem Básica, Universidade Federal de Minas Gerais, Belo Horizonte, MG, Brazil; 4 Departamento de Enfermagem, Hospital Universitário, USP, São Paulo, SP, Brazil; 5 Departamento de Orientação Profissional, Escola de Enfermagem, USP, São Paulo, SP, Brazil

**Keywords:** nursing, occupational health, job satisfaction, burnout, working environment, enfermagem, saúde do trabalhador, satisfação no emprego, esgotamento profissional, ambiente de trabalho

## Abstract

**Introduction:**

Characterized by high levels of emotional exhaustion and depersonalization and reduced professional accomplishment, burnout syndrome has been a major cause of psychic illness in nursing workers, with a serious impact on the quality of services and on patient safety

**Objectives:**

To analyze the correlation between organizational climate, job satisfaction, and burnout in nursing workers.

**Methods:**

This is a cross-sectional study with a sample of 534 Brazilian nursing workers. We used the Organizational Climate Scale for Health Organizations, the Job Satisfaction Questionnaire (S20/23), and the Maslach Burnout Inventory. An analytical descriptive analysis of the data was performed using relative and absolute frequencies, mean, standard deviation, minimum, maximum, and correlation test between the variables.

**Results:**

Organizational climate and job satisfaction were evaluated as regular. With regard to burnout, moderate levels of emotional exhaustion, low levels of depersonalization, and high levels of professional accomplishment were observed. A strong positive correlation was found between job satisfaction and organizational climate; in addition to a moderate negative correlation between emotional exhaustion and both organizational climate and job satisfaction, and a moderate negative correlation between depersonalization and job satisfaction.

**Conclusions:**

Organizational climate and job satisfaction had a negative correlation with burnout dimensions, representing possible protective factors.

## Abstract (pt)

**Introdução:**

Caracterizada por elevados níveis de exaustão emocional, despersonalização e diminuição da realização profissional, a síndrome de *burnout* tem sido uma grande causa de adoecimento psíquico em trabalhadores de enfermagem, com sérios impactos na qualidade dos serviços e na segurança do paciente.

**Objetivos:**

Analisar a correlação entre clima organizacional, satisfação no trabalho e *burnout* em trabalhadores de enfermagem.

**Métodos:**

Trata-se de um estudo transversal, realizado com uma amostra de 534 trabalhadores de enfermagem brasileiros. Utilizou-se a Escala de Clima Organizacional para Organizações de Saúde, o Questionário de Satisfação no Trabalho (S20/23) e o Inventário de Burnout de Maslach. Foi realizada uma análise analítica e descritiva dos dados por meio de frequências relativas e absolutas, média, desvio padrão, mínimo, máximo e teste de correlação entre as variáveis.

**Resultados:**

O clima organizacional e a satisfação no trabalho foram avaliados como regulares. Quanto ao *burnout,* observou-se moderada exaustão emocional, baixa despersonalização e elevada realização profissional. Verificou-se correlação positiva forte entre satisfação no trabalho e clima organizacional; correlação negativa moderada entre exaustão emocional e clima organizacional; correlação negativa moderada entre exaustão emocional e satisfação no trabalho; e correlação negativa moderada entre despersonalização e satisfação no Trabalho.

**Conclusões:**

Clima organizacional e satisfação no trabalho apresentaram correlação negativa com as dimensões de *burnout,* constituindo-se em possíveis fatores de proteção.

## INTRODUCTION

Given the important role of psychic distress on the disease process of nursing workers, burnout has been the focus of research in several countries, due to its impact not only on workers’ quality of life but also on work quality and, consequently on patient safety^1,2,3^

Conversely work can influence or act positively as a health promotion agent. Factors such as emotional intelligence, engagement, and well-being at work are considered indicators of improvement of nurses’ mental and occupational health.^4^ In this aspect, it is believed that job satisfaction and perception of a favorable organizational climate may have a protective role in the genesis of psychic illness.

Job satisfaction is defined as a complex subjective event that may vary for the same person and between individuals over time and according to circumstances.^5^ In nursing, “it is more often related to elements that the organization provides to workers, taking their expectations into account.”^6^

Organizational climate, “a temporary condition formed by meanings constructed by the individuals and that guide their decisions and actions in the organizational environment,” refers to the influence of working environment on workers’ behavior.^7^

Assuming that job satisfaction and favorable organizational climate are protective factors against burnout, it is believed that investigating these constructs is relevant to understand this relationship, so as to support the development of management strategies, with proactive organizational policies for the creation of a working environment that promotes health among nursing workers. Therefore, this study aimed to analyze the correlation between organizational climate, job satisfaction, and burnout in nursing workers.

## METHODS

This is an observational, analytical, cross-sectional and correlational study conducted in public/charitable health facilities of four municipalities in the Northern Coast of the state of São Paulo, Southeastern Brazil.

Before data collection, approval was granted from the managers of the health facilities and from the Research

Ethics Committee of Universidade de São Paulo (USP) School of Nursing (Opinion 912,483 of November 17^th^, 2014). Nursing workers were invited to participate in the study, and after giving their consent, they received an envelope containing the data collection instrument, and a day and time were scheduled for participants to return the questionnaire. Subsequently, the researcher returned to collect the completed questionnaires at least three times.

### STUDY PARTICIPANTS

The non-probabilistic sample of 534 nursing workers was obtained by convenience: 949 nursing workers received the instruments, accounting for 67.68% of the 1,402 nursing professionals who worked at four hospital institutions, one emergency medical service, one mobile emergency care service, and 51 basic health care facilities, from August 2015 to July 2016. Among the questionnaires returned, eight were excluded because over 20% of their items were incomplete.

Data collection was performed using the following instruments: a socio-demographic and professional characterization structured questionnaire; the Organizational Climate Scale for Health Organizations (Escala de Clima Organizacional para Organizações de Saúde, ECO OS),^7^ comprising 64 items distributed into seven factors; the Job Satisfaction Questionnaire (S20/23),^5^ consisting of 20 items and three factors; and the Maslach Burnout Inventory (MBl),^8^ composed of 22 items distributed into three dimensions.

### DATA ANALYSIS PROCEDURES

This study did not establish a cutoff value for the scales; analyses were conducted by calculating mean scores for each factor/dimension and also the overall mean of the items (overall score) for ECOOS and S20/23.

Data were inserted into an electronic database, with independent double entry. After correction of errors and inconsistencies, data were analyzed using the Statistical Package for the Social Sciences for Windows (SPSS®) software, version 22.0. Socio-demographic and professional characterization of nursing professionals, as well as ECOOS, S20/S23, and MBI-Human Service Survey (MBI-HSS) scores, were assessed with simple descriptive analysis. The associations between organizational climate, work satisfaction, and burnout syndrome dimensions were assessed using Pearson’s correlation. In order to identify and classify the magnitude of correlations, coefficients between 0.30 and 0.50 were considered as indicating moderate correlation, and those above 0.50 as indicating strong correlation.^9^ An analysis of internal consistency (Cronbach’s alpha) was conducted to confirm the psychometric qualities of the constructs: ECOOS (overall score: α = 0.97; 1- Leadership [LD]; α = 0.95; 2- Professional development [PD] α = 0.95; 3- Relationship and team spirit [ReTS] α = 0.92; 4-Relationship with the community [RC] α = 0.89; 5-Safety at work [SW] α = 0.94; 6- Strategy [ST] α = 0.92; 7-Compensation [COMP] α = 0.91), S 20/23 (overall score α = 0.93; 1- Satisfaction with hierarchical relationships [SHR] α = 0.93; 2- Satisfaction with the physical working environment [SPWE] α = 0.88; 3- Intrinsic satisfaction at work [ISW] α = 0.82), and MBI (1- Emotional exhaustion [EE] α = 0.89; 2- Depersonalization [DE] α = 0.66; 3- Professional accomplishment [PA] α = 0.74).

## RESULTS

### SOCIO-DEMOGRAPHIC AND PROFESSIONAL CHARACTERIZATION OF NURSING WORKERS

Of the 534 study participants, 90.45% were women, and more than a half was in a stable relationship (62.92%) and had children (69.29%). Most workers (92.5%) reported being the family provider or partially contributing to family income. Mean age of nursing professionals was 37.69 years, ranging from 19 to 67 years (SD = 8.88). Participants reported an average monthly personal net income of 2,136.72 Brazilian *reais* (BRL) (SD = 1,283.00), ranging from 600.00 to 10,000.00.

It was found that 52.24% reported working in hospital institutions, 42.51% in basic health facilities, 3% in technical-administrative and support departments, and 2.06% in pre-hospital care services. With regard to workers’ position, 72.28% were nursing assistants/technicians, 21.35% were clinical nurses, and 5.81% were in managerial positions.

### ANALYSIS OF ORGANIZATIONAL CLIMATE, JOB SATISFACTION, AND BURNOUT SYNDROME (TABLE 1)

**Table 1:** Distribution of perceived organizational climate, job satisfaction, and burnout in nursing workers

Factors/dimensions and overall scores	n	Mean	Standard deviation	Minimum	Maximum
ECOOS					
1- Leadership	517	3.60	1.01	1.00	5.00
2- Professional development	518	3.24	1.15	1.00	5.00
3- Relationship and team spirit	511	3.93	0.92	1.00	5.00
4- Relationship with the community	511	3.37	0.95	1.00	5.00
5- Safety at work	511	3.18	1.18	1.00	5.00
6- Strategy	509	2.86	1.09	1.00	5.00
7- Compensation	514	2.16	1.16	1.00	5.00
Overall score – organizational climate	527	3.32	0.78	1.00	5.00
S20/23					
1- Satisfaction with hierarchical relationships	522	3.40	0.98	1.00	5.00
2- Satisfaction with the physical working environment	523	3.27	1.13	1.00	5.00
3- Intrinsic satisfaction at work	523	3.61	0.93	1.00	5.00
Overall score – job satisfaction	523	3.41	0.88	1.17	5.00
MBI					
1- Emotional exhaustion	525	1.67	0.89	0.00	3.89
2- Depersonalization	524	0.86	0.76	0.00	3.00
3- Professional accomplishment	525	2.94	0.73	0.00	4.00

ECOOS = Organizational Climate Scale for Health Organizations (Escala de Clima Organizacional para Organizações de Saúde); MBI = Maslach Burnout Inventory; S20/23 = Job Satisfaction Questionnaire.

Table 1 shows that the mean overall score for organizational climate was 3.32; Factor 7-COMP was the one with the lowest mean, whereas Factor 3- ReTS was the one with the highest mean. Assessment of job satisfaction revealed that mean overall score was 3.41, ranging from 1.17 to 5.00. With regard to burnout dimensions, the highest mean was for PA.

### CORRELATIONS BETWEEN ORGANIZATIONAL CLIMATE, JOB SATISFACTION, AND BURNOUT SYNDROME (TABLE 2)

**Table 2:** Correlations between organizational climate, job satisfaction, and burnout syndrome dimensions in nursing workers

Overall score/dimensions	Organizational climate	Job satisfaction
Job satisfaction	0.673†	1
Burnout		
Emotional exhaustion	−0.408†	−0.457†
Depersonalization	−0.298†	−0.319†
Professional accomplishment	0.193†	0.146†

Test: Pearson’s correlation.

*Significance level 0.05.

† Significance level 0.01.

As shown in Table 2, a strong positive correlation was observed between job satisfaction and organizational climate, in addition to a moderate negative correlation between EE and both organizational climate and job satisfaction, and a moderate negative correlation between DE and job satisfaction.

Table 3 presents the correlations between the dimensions of each construct, in order for readers to better visualize the impact of each item of the scales.

**Table 3:** Correlations between factors/dimensions of organizational climate, job satisfaction, and burnout syndrome in nursing workers

Variables	Job satisfaction			Burnout		
	Factor 1 SHR	Factor 2 SPWE	Factor 3 ISW	EE	DE	PA
**Organizational climate**						
Factor 1- LD	0.634*	0.320*	0.406*	−0.278*	−0.247*	0.163*
Factor 2- PD	0.404*	0.266*	0.347*	−0.255*	−0.151*	0.112†
Factor 3- ReTS	0.446*	0.286*	0.352*	−0.308*	−0.232*	0.119*
Factor 4- RC	0.460*	0.423*	0.367*	−0.396*	−0.337*	0.146*
Factor 5- ST	0.506*	0.576*	0.415*	−0.343*	−0.273*	0.156*
Factor 6- SW	0.475*	0.375*	0.384*	−0.308*	−0.177*	0.144*
Factor 7-COMP	0.324*	0.332*	0.348*	−0.265*	−0.087†	−0.018
**Burnout**						
EE	−0.410*	−0.337*	−0.449*			
DE	−0.311*	−0.238*	−0.243*			
PA	0.132*	0.062	0.207*			

Test: Pearson’s correlation.

* Significance level of 0.01.

† Significance level of 0.05.

COMP = compensation; DE = depersonalization; EE = emotional exhaustion; ISW = intrinsic satisfaction at work; LD = leadership; PA = professional accomplishment; PD = professional development; RC = relationship with the community; ReTS = relationship and team spirit; SPWE = satisfaction with the physical working environment; SHR = satisfaction with hierarchical relationships; ST = strategy; SW = safety at work.

## DISCUSSION

Organizational climate was assessed as regular by nursing workers (overall mean score [*x* = 3.32], considering a scale from 1 to 5). The evaluation of each factor in the scale revealed that COMP and ST were the critical nodes of organizational climate in the institutions where the research was conducted, since they received the lowest scores. Conversely, ReTS and LD stood out as the factors that strengthened organizational climate in the institutions investigated.

Participants reported an average monthly personal net income of 2,136.72 BRL (DP 1,283.00), approximately 403.69 USD, with a minimum income of 600.00 BRL, showing the low wages paid in the market for nursing workers in the region where the study was developed. The variability in personal income is also associated with worker’s position in the institution: nursing assistants/technicians usually earn lower wages compared to clinical nurses or those in managerial positions. Dissatisfaction with wages is in line with a Brazilian study with nursing assistants and technicians showing that low wages are one of the elements contributing to job insecurity.^10^

The ST factor, which received the second lowest mean score among all ECOOS factors, corresponds to the components of institution’s strategic planning, including workers’ participation in the process of strategic planning development, presence of a change management plan for growth and development, and encouragement to participate in the change process.^7^

In Brazil, most health organizations lack spaces that allow workers to be heard and included in the decisionmaking process with greater autonomy and appreciation. Conversely, local managers face considerable difficulties, especially in public services, due to limited autonomy in problem solving, such as multiple employment relationships, lack of material and human resources, and reduced funding. Therefore, it is necessary to make innovations to the management model covering all organizational spheres.^11^

Positive results for LD are consistent with those of another Brazilian study on organizational climate.^12^ It is worth highlighting the positive relationship between managers and the work team members, which increases commitment and motivation for the development of work activities, bringing benefits both to workers and to the institution.^13^

Additionally, it is consensus that recognition and appreciation at work are related to job satisfaction and that their lack leads to suffering, with consequences to workers’ health.^13,14^

With regard to SW, the literature points out that this indicator can be improved by incorporating safety strategies that promote greater adherence to safety rules and, consequently, favor a safety climate, safe care, and bonding between workers and managers. The perception of workers and managers on safety reflects the institutional safety climate; this climate in turn is related to organizational policies, to managerial decisions, and to the implementation of safety programs, collaborating in the prevention of work-related diseases.^15,16^

Results for job satisfaction revealed that, according to the overall mean score for this variable, nursing workers presented medium levels of satisfaction (*x̅* = 3.40, on a scale from 1 to 5), a result similar to that of studies conducted with nursing professionals in Spain and Brazil.^17,18^

In relation to the individual analysis of job satisfaction factors, SPWE received the lowest scores, and the main problems identified were climate control at the workplace, environment and physical space, and workplace ventilation. In the literature, better working environmental conditions have been associated with increased job satisfaction.^19^ However, investing on climate control, ventilation, and appropriate physical workspace will promote better levels of job satisfaction among workers from the investigated scenario.

With regard to burnout syndrome, there was great variability in the national and international literature when comparing the levels of each burnout syndrome in nursing workers.^17,20,21,22^ Considering that the scores of the scale used ranged from 0 to 4, despite low levels of DE (*x* = 0.86) and high levels of PA (*x* = 2.94), mean levels of EE (*x* = 1.67) were observed, indicating a preventive factor for intervention, because EE is considered the first stage of burnout syndrome, since it leads to DE and may interfere with PA.^23^

It is worth highlighting that EE can impair workers’ concentration and judgment, thus affecting patient safety. Research conducted with nurse managers in Brazil revealed that they experienced anguish and many conflicts due to their perception that the quality of the work performed by many professionals is impaired by psychic burnout.^24^

In addition to the consequences of burnout to the health of nursing worker’s, this condition raises concern because it is a recognized predictor of suicide risk, being associated with EE, lack of professional recognition and professional encouragement, and low personal accomplishment, which may result in feelings of incompetence, failure, despair, hopelessness, depression, and suicidal ideation.^25^

The results obtained allow us to state that this study supports the hypothesis of a positive association between job satisfaction and organizational climate as protective factors against burnout syndrome.

Workers who expressed greater levels of overall job satisfaction also attributed higher scores to overall organizational climate, showing the strong correlation between these two constructs. In the analysis of the association between the factors of the two scales, there was a significant positive association between all organizational climate factors with SHR and ISW. SPWE was also positive correlated with LD, RC, SW, ST, and COMP. The negative correlation between overall job satisfaction and EE corroborates findings from other studies conducted in different scenarios.^17,26,27^

In the individual analysis of the association between the construct factors, it was found that the lower the scores for SHR, SPWE, and SIW, the higher the score for EE. These dados partially corroborate those obtained by Sá et al.,^28^ whose research with nursing workers found that SPWE and ISW exert a negative influence on EE, whereas SHR showed no influence on EE.

In the study sample, SHR decreases as the likelihood of DE increases. Therefore, supervision style and workers’ perception about their relationship with managers have an influence on the development of burnout syndrome, bringing consequences to workers’ psychic health and to the individuals receiving care from these workers.

With regard to the positive association between overall job satisfaction and DE, other studies also observed statistically significant negative correlations between these variables.^26,27^ It is believed that work dissatisfaction leads workers to distancing-themselves from the recipients of their work as a way to deal with discontent.^27^

Despite the growing interest in investigating organizational climate, there are still few studies with nursing workers that evaluated the relationship between organizational climate and burnout. In the present research, nursing workers with better perceived organizational climate were those with the lowest levels of EE, a finding that is line with that found in a study with workers from a hospital in the country region of the state of São Paulo, Brazil.^1^

An individual analysis of the association between burnout and organizational climate factors revealed that nursing workers with higher perceived scores for ReTS, RC, SW, and ST had lower EE scores; likewise, those who reported a positive relationship with the community showed lower levels of DE.

However, one can infer that a perceived favorable organizational climate makes nursing workers less susceptible to EE and thus less exposed to burnout.

### CONTRIBUTION TO PRACTICE

Once an association between organizational climate, job satisfaction, and burnout syndrome was observed, it is crucial to intervene in this reality, since burnout is influenced by the organization of the work process, bringing consequences to worker’s health, patient safety, and organizational success.

Based on these results, one can assume that identification of perceived organizational climate and level of job satisfaction, as well as analysis of critical nodes and subsequent implementation of remedial policies for changing the organizational context, may reduce the occurrence of burnout syndrome among nursing workers. Therefore, improving organizational climate not only improves satisfaction but also acts as a protective element against burnout.

### STUDY LIMITATIONS

The limitations of this study arise from its cross-sectional design, which permits to analyze the associations between variables but not allow for establishing causal relationships; furthermore, the results obtained refer to a specific geographic region. Another relevant factor is that participation in the study was limited to nursing professionals who were actively working, with the exclusion of those who were on medical leave, due to the difficulty in reaching these workers.

## CONCLUSIONS

Evaluating organizational climate and job satisfaction and correlating them with burnout syndrome enabled to, based on the individual analysis of each dimension, recognize the risk factors that make nursing workers more vulnerable to the development of this syndrome. Findings indicate that organizational climate has a strong positive correlation with job satisfaction and that both organizational climate and job satisfaction have an impact on EE, showing a moderate negative correlation and representing possible protective factors. Job satisfaction has also an impact on DE, since the greater job satisfaction, the lower DE.

The results of this study may help in the management of nursing workers and in reducing their burnout levels, which will have repercussions on workers’ health and on the quality of the services provided.
